# Serial Diffusion Tensor Imaging of the Optic Radiations after Acute Optic Neuritis

**DOI:** 10.1155/2016/2764538

**Published:** 2016-07-31

**Authors:** Scott C. Kolbe, Anneke van der Walt, Helmut Butzkueven, Alexander Klistorner, Gary F. Egan, Trevor J. Kilpatrick

**Affiliations:** ^1^Department of Anatomy and Neuroscience, University of Melbourne, Parkville, VIC 3010, Australia; ^2^Florey Institute of Neuroscience and Mental Health, Parkville, VIC 3010, Australia; ^3^Department of Neurology, Royal Melbourne Hospital, Parkville, VIC 3052, Australia; ^4^Department of Medicine, University of Melbourne, Parkville, VIC 3010, Australia; ^5^Save Sight Institute, University of Sydney, Sydney, NSW 2000, Australia; ^6^Monash Biomedical Imaging, Monash University, Clayton, VIC 3800, Australia

## Abstract

Previous studies have reported diffusion tensor imaging (DTI) changes within the optic radiations of patients after optic neuritis (ON). We aimed to study optic radiation DTI changes over 12 months following acute ON and to study correlations between DTI parameters and damage to the optic nerve and primary visual cortex (V1). We measured DTI parameters [fractional anisotropy (FA), axial diffusivity (AD), radial diffusivity (RD), and mean diffusivity (MD)] from the optic radiations of 38 acute ON patients at presentation and 6 and 12 months after acute ON. In addition, we measured retinal nerve fibre layer thickness, visual evoked potential amplitude, optic radiation lesion load, and V1 thickness. At baseline, FA was reduced and RD and MD were increased compared to control. Over 12 months, FA reduced in patients at an average rate of −2.6% per annum (control = −0.51%; *p* = 0.006). Change in FA, RD, and MD correlated with V1 thinning over 12 months (FA: *R* = 0.450, *p* = 0.006; RD: *R* = −0.428, *p* = 0.009; MD: *R* = −0.365, *p* = 0.029). In patients with no optic radiation lesions, AD significantly correlated with RNFL thinning at 12 months (*R* = 0.489, *p* = 0.039). In conclusion, DTI can detect optic radiation changes over 12 months following acute ON that correlate with optic nerve and V1 damage.

## 1. Introduction

In later stages of multiple sclerosis (MS), patients can develop a “secondary progressive” (SPMS) disease phenotype that is not associated with neuroinflammatory relapses but rather with axonal injury and primary neuronal pathologies. SPMS is largely untreatable because disease modifying therapies for MS act on peripheral inflammatory cells. Therefore, to develop new treatments for secondary progressive MS, it is critical to identify the disease mechanisms underlying the transition from relapsing-remitting to SPMS.

Several candidate neurodegenerative processes have been proposed, including excitotoxicity and transsynaptic degeneration [1]. Transsynaptic degeneration occurs when a neuron loses afferent input or efferent targets. The precise molecular mechanisms of transsynaptic degeneration continue to be investigated. Nonetheless, given that multifocal sites of neuroinflammatory injury in MS can transect axons throughout the central nervous system, transsynaptic degenerative processes could be responsible for nonlesional, secondary neurodegeneration in neural circuits in the brain.

Studies of the visual pathway in MS and optic neuritis (ON) provide an appropriate model brain network with which to study transsynaptic degeneration* in vivo*. Animal model studies [2] and human neuroimaging evidence consistently demonstrate that transsynaptic degeneration occurs in the optic radiations after neuronal injury to the afferent visual system in conditions such as Leber's hereditary optic neuropathy [3], glaucoma [4], neuromyelitis optica [5], and amblyopia [6]. Similarly ON is associated with optic nerve axonal degeneration that is evident as retinal nerve fibre layer (RNFL) thinning using optical coherence tomography (OCT) and diminished visual evoked potentials (VEP). Recent work by our group and others has demonstrated that the degree of RNFL thinning and VEP attenuation in the eye affected by ON is associated with anterograde microstructural changes to the optic radiations and visual cortex using diffusion-weighted and magnetisation transfer MRI techniques [7–10]. In particular, our group reported that VEP attenuation was associated with the reduction in axial diffusivity (AD) derived from diffusion tensor imaging (DTI). AD has been shown to correlate histologically with axonal degeneration in an animal model of MS [11]. In contrast to our previous findings, a recent paper by Raz and colleagues [12] reported increased radial diffusivity (RD), decreased fractional anisotropy (FA), and no change in AD in the optic radiations of 17 patients with a history of acute ON. Importantly the RD and FA changes correlated with lesion volume in the radiations rather than optic nerve injury leading the authors to conclude that transsynaptic degeneration was not detectable, yet it could have been masked by Wallerian degeneration due to lesions in the radiations.

All previous studies assessing postgeniculate alterations after acute ON have been cross-sectional rather than longitudinal in design. Therefore, the timing of the observed DTI changes with respect to acute ON has yet to be characterised. The present study involved serial DTI assessments of the optic radiations in 38 patients with acute ON. We aimed to determine whether DTI changes could be detected within the first 12 months after acute presentation and the degree to which such changes were associated with neurodegenerative changes in the optic nerve and cortex.

## 2. Materials and Methods

Forty-one adults presenting within two weeks of symptom onset with a first episode of unilateral optic neuritis were recruited consecutively between 2008 and 2010 from a tertiary ophthalmology hospital (Royal Victorian Eye and Ear Hospital). ON was diagnosed by identical clinical criteria used for the optic neuritis treatment trial [13]. Thirty patients were treated acutely with intravenous methylprednisolone. Two patients withdrew from the study prior to 6-month testing. One patient was excluded due to recurrent ON. Two patients missed 6-month testing. The final group included in subsequent analyses included 38 patients (26 F/12 M, median [IQR] age = 37 [28.5 to 41.75] years). All patients had unilateral optic nerve lesions observable on T2-weighted MRI (reported previously in [14]) and at least two T2-hyperintense brain lesions on baseline MRI scans indicating a high risk of subsequent development of MS (87% over the ensuing ten years) [15]. Patients were excluded if they had previously been diagnosed with an alternative neurological or ophthalmological disease or presented with further episodes of ON during the study. Patients were not excluded if they met MRI-based criteria for diagnosis with MS [16]. Of the 38 patients studied, six were diagnosed with relapsing-remitting MS (RRMS) at baseline and further 13 were diagnosed with RRMS during the 12 months of the study based on presentation with a second clinical relapse. At 12 months, 14 patients were receiving disease modifying treatment for MS (interferon beta 1a: 7; interferon beta 1b: 2; glatiramer acetate: 4; natalizumab: 1).

Twenty-three healthy subjects (15 F/7 M, median [IQR] age = 36 [32.5 to 42.5] years) were scanned twice, 18 months apart using the same MRI sequences to assess healthy longitudinal differences in optic radiation DTI.

This study was conducted in accordance with the Declaration of Helsinki and was approved by the Human Research Ethics Committees of the Royal Victorian Eye and Ear Hospital and the Royal Melbourne Hospital. All study participants provided voluntary, written consent.

### 2.1. Magnetic Resonance Imaging

Subjects were imaged using a Siemens 3T Trio MRI system with a 32-channel receiver head coil. Each scanning session included a spin-echo echo-planar imaging diffusion-weighted imaging sequence (TR = 8200 ms; TE = 89 ms; voxel dimensions = 2.0 × 2.0 × 2.0 mm^3^; GRAPPA parallel speed factor = 2; 10 non-diffusion-weighted volumes and 60 noncollinearly applied diffusion gradient-applied volumes with gradient* b*-value = 1200 s/mm^2^; acquisition time = 9 m 59 s), a 3D T1-weighted volumetric sequence (TR = 1900 ms, TE = 2.63 ms, TI = 900 ms, flip angle = 9°, voxel dimensions = 0.8 × 0.8 × 0.8 mm^3^, GRAPPA parallel speed factor = 2, and acquisition time = 5 m 25 s), and a 3D T2-weighted fluid-attenuated inversion recovery (FLAIR) sequence (TR = 5000 ms, TE = 436 ms, TI = 2100 ms, flip angle = 120°, voxel dimensions = 0.9 × 0.9 × 0.9 mm^3^, GRAPPA parallel speed factor = 2, and acquisition time = 7 m 37 s). Lesions were defined on each patient's FLAIR image using a semiautomated local thresholding technique [17] by an experienced observer (SK).

### 2.2. DTI Preprocessing, DTI Template Construction, and Optic Radiation ROI Mapping

Whole brain diffusion-weighted images were eddy-current corrected and realigned using a linear registration algorithm (FLIRT, FSL) [18]. The diffusion tensor was calculated using FSL diffusion toolkit [19]. In order to perform region of interest analyses on subject-specific optic radiations, we first created a study-specific DTI atlas in MNI-152 space using a diffusion tensor image registration toolkit (DTI-TK) [20]. The atlas was constructed from a single randomly chosen DTI study from 20 randomly selected subjects (10 MS and 10 controls) using the following procedure. Firstly, an initial atlas estimate was obtained by linearly registering each DTI dataset to the ixi-adult template publically available with the DTI-TK software (https://www.nitrc.org/frs/download.php/5519/ixi_adult_template_v1.1.tgz) and averaging the resulting coregistered images. Next, each subject's DTI data were affinely registered to the initial template and averaged to create a second-pass template. Thirdly, each subject's DTI data were registered to the second-pass template using the affine and a deformable nonlinear registration. The resulting images were averaged and this process was repeated six times to iteratively improve the template.

Once the final template was calculated, all data (all subjects and all time-points) were registered to the template using a combined affine and deformable registration. Fractional anisotropy (FA), axial diffusivity (AD), radial diffusivity (RD), and mean diffusivity (MD) maps were calculated from the normalised DTI data for each subject.

**Figure 1:**
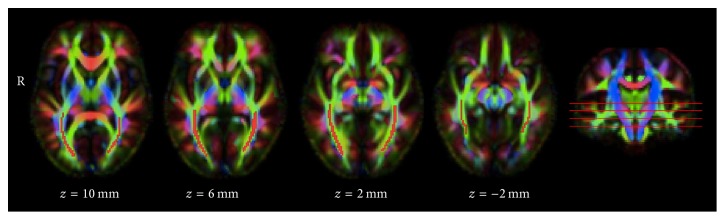
Diffusion tensor imaging template generated from 10 random controls and 10 random patients using DTI-TK. The optic radiation region of interest used to calculate DTI parameters is shown in red against the green background of the optic radiations.

Manual regions of interest were drawn on the template in the centre of the left and right optic radiations individually by an expert observer (SK) guided by previously calculated maps based on tractography [7] (Figure 1). Manual ROIs were chosen over tractography ROIs to ensure that the centre of the tract was specifically selected. Each scan for each subject was carefully checked for registration quality to ensure that the ROI captured the centre of the tract. Lesion masks were linearly transformed to diffusion space using an affine registration algorithm (FLIRT) [18] and lesion voxels were ignored from ROI mean DTI calculations.

### 2.3. Primary Visual Cortex Thickness

T1-weighted volumetric images were processed using FreeSurfer 5.3.0 (http://freesurfer.net/) using the standard processing pipeline [21]. Thickness of primary visual cortex (area 17) was obtained from the standard FreeSurfer output.

### 2.4. Visual Function Testing and Retinal Imaging

Patients were tested using Accumap™ (ObjectiVision, software: Opera, Sydney, Australia) to obtain mfVEP with a previously described testing procedure. Recordings from 58 sectors of the visual field were obtained using four electrodes placed over the inion on the rear of the skull. Eyes were individually stimulated for 10 to 12 runs until a sufficient signal to noise ratio (SNR) was reached. Mean amplitude from 6- and 12-month time-points was calculated per visual field sector and then per eye as a whole for affected and unaffected eyes. VEP amplitude attenuation in affected eyes was represented as an interocular percentage difference from unaffected for subsequent analyses to remove intersubject variability.

OCT was performed using a time-domain OCT-3 scanner (Stratus™, software version 3.0, Carl Zeiss Meditec Inc.) using the Fast RNFL protocol consisting of three circular 3.4 mm diameter scans centred on the optic disc. Signal strength of seven or more was deemed acceptable. RNFL thickness was measured from 6- and 12-month time-points for the affected and unaffected eyes. RNFL thinning in affected eyes was represented as an interocular percentage difference from unaffected for subsequent analyses to remove intersubject variability.

### 2.5. Statistical Analyses

Average FA, AD, RD, and MD were calculated for left and right optic radiation ROIs for each subject at each time-point. Left and right data were compared using paired* t*-tests. No significant interhemispheric differences in DTI parameters were found for either patients or controls (see Supplementary Table 1 in Supplementary Material available online at http://dx.doi.org/10.1155/2016/2764538) so left and right values were averaged to obtain a single value for each DTI parameter for each subject and time-point.

Patient baseline DTI parameters were compared to control using unpaired two-sided* t*-tests. For patients and controls, annualised percentage changes in DTI parameters and V1 thickness were calculated from all imaging time-points. Annualised optic radiation DTI change was compared between patients and controls using unpaired two-sided* t*-tests.

To assess the sources of DTI change in the optic radiations, annualised change in DTI parameters was compared to 12-month RNFL thickness and mfVEP amplitude outcomes, annualised change in V1 thickness, and lesion volume (logarithmically transformed) using partial correlation analyses, corrected for age. All statistical analyses were performed using SPSS® 21.0 (IBM® Corporation).

## 3. Results

Demographic and disease parameters for patients and controls are described in Table 1. At baseline, patients showed a cerebral lesion load of between 0 and 38 mL with a median of one newly appearing lesion during the study. From the cohort of 38 patients, 17 displayed no optic radiation lesions. The remaining 19 patients had a median (range) optic radiation lesion volume of 0.61 (range = 0 to 21.3) mL.

At baseline, optic radiation FA (*p* = 0.035), RD (*p* = 0.021), and MD (*p* = 0.049) were significantly different between patients and controls (Table 2). During the subsequent 12 months, the mean (±SD) annualised rate of change in FA in patients (−2.6 ± 0.53%) exceeded that of healthy controls (−0.51 ± 0.50%, *p* = 0.006). No other DTI parameters displayed a significant longitudinal change; however, there was a trend towards a significant increase in RD in patients (2.82 ± 0.61%) compared to controls (1.30 ± 0.48%, *p* = 0.06).

In patients, annualised changes in optic radiation FA (*R* = 0.450, *p* = 0.006), RD (*R* = −0.428, *p* = 0.009), and MD (*R* = −0.365, *p* = 0.029) were all associated with the annualised rate of change in V1 cortical thickness (Table 3). However, none of these DTI parameters were associated with thinning of the RNFL or attenuation of VEP. In contrast, reduced AD in patients with no optic radiation lesions correlated with RNFL thinning (*R* = 0.489, *p* = 0.047) and showed a trend towards correlation with VEP attenuation (*R* = 0.481, *p* = 0.06). There were no significant correlations between any optic radiation DTI parameters and the volume of lesions in the optic radiations.

## 4. Discussion

This study assessed the optic radiations of 38 patients serially over 12 months following acute ON using DTI. At baseline, patients displayed significant change in FA, RD, and MD compared to controls, indicating preexisting pathology prior to initial presentation. Over the subsequent 12 months, FA reduced significantly in patients compared to controls. Longitudinal change in FA, RD, and MD significantly correlated with V1 thinning. Cortical thinning is believed to be primarily associated with neurodegeneration, so the decline in FA and the increases in RD and MD most likely reflect primary neurodegeneration of the optic radiations. White matter lesions within the optic radiations provide a potential source of such neurodegeneration, given previous histological observations of significant axonal transection in MS lesions [22]. However, in patients with optic radiation lesions (*n* = 17), coefficients for correlation tests between optic radiation lesion volume and NAWM DTI parameters were low (~0.3) indicating that larger lesion volumes did not directly result in greater neurodegeneration in those patients. Given the use of high resolution 3D FLAIR scans for lesion detection, white matter lesions could be accurately delineated. However, a second potential source of optic radiation neurodegeneration is cortical lesions in V1. The detection of such lesions was not possible in the present study because cortical lesions cannot be reliably detected on standard FLAIR T2 imaging but require a second inversion pulse to suppress grey matter signals [23]. Therefore, cortical lesions could accounted for the lack of correlation between NAWM DTI parameters and white matter lesion volume.

In patients with no optic radiation lesions (*n* = 19), optic nerve axonal loss was associated with reduced optic nerve AD. This result confirms our previous cross-sectional observations in patients with a history of unilateral optic neuritis suggesting that optic radiation AD reduction is a marker for anterograde transsynaptic degeneration [7]. Interestingly, AD reduction was not associated with V1 thinning. Therefore, AD reduction might be a specific marker of transsynaptic rather than Wallerian degeneration. These two pathologies differ at the microscopic scale that DTI is sensitive to. Wallerian degeneration is characterised by axonal transection followed by retrograde axonal degeneration leading ultimately to neuronal degeneration. In contrast, anterograde transsynaptic degeneration is characterised by neuronal atrophy and ultimately apoptosis [24, 25]. Reduced AD has been observed in the context of acute axonal injury in animal models [11, 26–28] and humans [29–31]. The precise biophysical mechanisms leading to AD reduction remain uncertain but could include axonal loss and atrophy [11] leading to a bulk reduction of the intra-axonal volume and associated anisotropic diffusion profile.

It is likely that transsynaptic degeneration occurs in all regions of the CNS affected by acute inflammatory axonal degeneration. However, translation of AD as a marker for transsynaptic degeneration in many brain white matter tracts is problematic. Inflammatory pathology within a white matter pathway could mask AD reduction by causing a general increase in diffusivity due to loss of tissue density and subsequent increase in extracellular isotropic diffusion. Moreover, whilst the diffusion tensor model is sufficient for modelling diffusion anisotropy in collinear structures such as the body of the optic radiations, the majority of the cerebral white matter contains more complex crossing or kissing fibre architectures [32]. Novel diffusion imaging techniques are currently being developed that aim to measure intra-axonal diffusion in the presence of complex axonal architecture [33, 34]. Such methods, once validated, are likely to become important radiological tools for mapping axonal degeneration in complex white matter in neurodegenerative diseases such as MS.

## 5. Conclusions

Our results indicate that the optic radiations undergo microstructural changes in the first year after acute ON that reflect primary (associated with V1 cortical thinning) and secondary (associated with optic nerve axonal loss) neurodegeneration. Diffusion tensor imaging is sensitive to these changes and could be used to identify patients with more severe neurodegenerative disease burden.

## Supplementary Material

The supplementary table includes summary statistics for left and right hemisphere optic radiation DTI parameters.

## Figures and Tables

**Table 1:** Demographic and disease summary data for acute ON patients and healthy controls.

	Patients (*n* = 38)	Controls (*n* = 23)
Median age (range) years	37 (18 to 50)	36 (24 to 49)
Sex distribution	26 F/12 M	15 F/7 M
CIS/MS at 12 months	19/19	—
Median lesion volume (range) mL	0.54 (0 to 38.1)	—
Median number of new lesions	1 (0 to 4)	—
Median OR lesion volume (range) mL	0.06 (0 to 21.3)	—

**Table 2:** Average (±SD) baseline and annualised change in optic radiation fractional anisotropy (FA), axial diffusivity (AD), radial diffusivity (RD), and mean diffusivity (MD) for healthy controls (HC) and optic neuritis (ON) groups.

Parameter	Group	Baseline		Annualised change (%)	
FA	HC	0.53 ± 0.03		−0.51 ± 0.50	
	ON	0.51 ± 0.04	**p** = 0.035	−2.60 ± 0.53	**p** = 0.006

AD (10^−3^ mm^2^s^−1^)	HC	1.25 ± 0.05		0.71 ± 0.33	
	ON	1.26 ± 0.06	*p* = 0.41	0.08 ± 0.26	*p* = 0.15

RD (10^−3^ mm^2^s^−1^)	HC	0.50 ± 0.03		1.30 ± 0.48	
	ON	0.53 ± 0.05	**p** = 0.021	2.82 ± 0.61	*p* = 0.06

MD (10^−3^ mm^2^s^−1^)	HC	0.75 ± 0.03		0.96 ± 0.31	
	ON	0.77 ± 0.05	**p** = 0.049	1.35 ± 0.37	*p* = 0.43

**Table 3:** Pearson *R* coefficients and associated probability values for correlations between annualised rate of change in optic radiation DTI parameters and annual change in RNFLT, mfVEP, and primary visual cortex (V1) thickness and logarithmically transformed baseline lesion volume in the optic radiations (OR). For correlations with RNFLT and mfVEP change, only patients without OR lesions were included. For correlations with log OR lesion volume, only patients with OR lesions were included.

		Annualised change in DTI parameters			
		FA	AD	RD	MD
V1 thickness change (*n* = 37)	*R*	**0.450**	−0.082	**−0.428**	**−0.365**
	*p*	**0.006**	0.63	**0.009**	**0.029**

RNFLT change (*n* = 17)	*R*	0.115	**0.489**	0.174	0.348
	*p*	0.66	**0.047**	0.50	0.17

mfVEP amplitude change (*n* = 17)	*R*	0.301	0.481	−0.061	0.161
	*p*	0.25	0.06	0.82	0.55

Baseline OR lesion volume (*n* = 19)	*R*	−0.321	0.257	0.31	0.31
	*p*	0.19	0.30	0.21	0.21
