# Supplementary material for: Serial Diffusion Tensor Imaging of the Optic Radiations after Acute Optic Neuritis
Source: J Ophthalmol. 2016 Jul 31;2016:2764538. doi: 10.1155/2016/2764538 (PMC4983385; doi:10.1155/2016/2764538)
Supplement: Supplementary file 1 — The supplementary table includes summary statistics for left and right hemisphere optic radiation DTI parameters. [file 2764538.f1.pdf]

Supplementary Table 1. Means and standard deviations (SD) for DTI parameters measured from left and right optic radiation ROIs and p-values for paired t-tests between left and right.

		Control				Patient			
		FA	AD	RD	MD	FA	AD	RD	MD
Left	Mean	0.53	1.26	0.51	0.76	0.50	1.26	0.54	0.78
	SD	0.028	0.048	0.029	0.027	0.039	0.064	0.061	0.057
Right	Mean	0.53	1.26	0.51	0.76	0.50	1.27	0.54	0.78
	SD	0.036	0.066	0.032	0.034	0.039	0.077	0.062	0.062
	p	0.54	0.58	0.41	0.79	0.84	0.39	0.81	0.59
